# Risk Factors for Recurrence of Intracranial Aneurysm After Coil Embolization: A Meta-Analysis

**DOI:** 10.3389/fneur.2022.869880

**Published:** 2022-07-22

**Authors:** Ji Jin, Geng Guo, Yeqing Ren, Biao Yang, Yongqiang Wu, Shule Wang, Yanqi Sun, Xiaogang Wang, Yuxiao Wang, Jianzhong Zheng

**Affiliations:** ^1^School of Public Health, Shanxi Medical University, Taiyuan, China; ^2^Department of Neurosurgery, The First Hospital, Shanxi Medical University, Taiyuan, China

**Keywords:** intracranial aneurysm, coil embolization, recurrence, meta-analysis, risk factors

## Abstract

Intracranial aneurysm is a severe cerebral disorder involving complicated risk factors and endovascular coiling is a common therapeutic selection for intracranial aneurysm. The recurrence is a clinical challenge in intracranial aneurysms after coil embolization. With this study, we provided a meta-analysis of the risk factors for the recurrence of intracranial aneurysm after coil embolization. Nine studies were included with a total of 1,270 studies that were retrieved from the database. The sample size of patients with intracranial aneurysms ranged from 241 to 3,530, and a total of 9,532 patients were included in the present meta-analysis. The intracranial aneurysms that occurred in middle cerebral artery (MCA) (OR = 1.09, 95% CI: 1.03–1.16, *P* = 0.0045) and posterior circulation (OR = 2.01, 95% CI: 1.55–2.60, *P* = 0.000) presented the significantly higher risk of recurrence after coil embolization. Meanwhile, intracranial aneurysms of size > 7 mm (OR = 5.38, 95%CI: 3.76–7.70, *P* = 0.000) had a significantly higher risk of recurrence after coil embolization. Moreover, ruptured aneurysm (OR = 2.86, 95% CI: 2.02–4.04, *P* = 0.000) and subarachnoid hemorrhage (SAH) (OR = 1.57, 95% CI: 1.20–2.06, *P* = 0.001) was positively correlated with the risk of recurrence after coil embolization. In conclusion, this meta-analysis identified the characteristics of intracranial aneurysms with MCA, posterior circulation, size > 7 mm, ruptured aneurysm, and SAH as the risk factors of recurrence after coil embolization for intracranial aneurysms.

## Introduction

The cerebral arterial aneurysm is a severe and prevalent disorder, which is a leading cause of sudden neurological disability secondary to rupture (1, 2). Intracranial aneurysms are among the most popular non-traumatic risk factors of subarachnoid hemorrhage (SAH) with an increasing incidence, resulting in a heavy economic and social burden globally (3, 4). Even though the patients accept therapy, many cases will ultimately die or suffer from a cognitive disability or severe neurological (5, 6). The treatment of intracranial aneurysms is associated with multiple unexpected risk factors (7, 8). Endovascular coiling has become a prevalent therapeutic selection for intracranial aneurysm patients in many hospitals (9, 10). Nevertheless, the recurrence of the intracranial aneurysm treated by endovascular coiling is still a crucial problem in the clinic (11). Numerous risk factors are involved in the events of recurrence and rebleeding (12, 13), which are poorly understood.

In this study, we were interested in the exploration of the correlation of risk factors, such as gender, smoking, posterior circulation, anterior cerebral artery (ACA), interior carotid artery (ICA), middle cerebral artery (MCA), aneurysms of size, aneurysms of the neck, ruptured aneurysm, and SAH, with the recurrence of intracranial aneurysms after coil embolization.

## Materials and Methods

### Literature Inclusion and Exclusion Criteria

The inclusion criteria were as follows: the study type is a retrospective study; the language is limited to English.

Exclusion criteria: duplicate publication; research without full text, research without the needed information of this study, incomplete information, or inability to conduct data extraction; animal experiments; reviews and systematic reviews.

### Search Strategy

In this meta-analysis, we searched Pubmed, Embase, and Cochrane Library from the establishment of the database to December 2020. The search terms are mainly: “Intracranial Aneurysm” “Brain Aneurysm” “Anterior Communicating Artery Aneurysm” “Basilar Artery Aneurysm” “Cerebral Aneurysm” and “Coil embolization” “recurrence”. Keywords were combined with Boolean operators to increase search sensitivity and specificity.

### Literature Screening and Data Extraction

The literature search, screening, and information extraction were all independently completed by two researchers. When there were doubts or disagreements, the decision was made after discussion or consultation with a third party. The data extraction included the author, year, study area, research type, number of cases, and the OR and 95%CI of age, smoking, posterior circulation, ACA (anterior cerebral artery), ICA (interior carotid artery), MCA (middle cerebral artery), Ruptured aneurysm, SHA (subarachnoid hemorrhage), aneurysm size > 7mm and aneurysm neck > 4mm for the prediction of recurrence after coil embolization for intracranial aneurysms. Aneurysm recurrence was defined as inflow into a previously completely occluded aneurysm or growth of an incompletely occluded aneurysm (aneurysm recanalization) (14).

### Literature Quality Assessment

Two authors (GENG GUO and JIANZHONG ZHENG) independently conducted literature quality evaluations using the NOS (Newcastle-Ottawa Scale) for the retrospective study. When the opinions are inconsistent, it is decided through discussion or consultation with a third person. The meta-analysis was performed based on the related items of the Preferred Reporting Items for Systematic Reviews and Meta-analysis statement (PRISMA statement).

### Data Synthesis and Statistical Analysis

Following eligibility verification, data about the risk factors, such as gender, smoking, posterior circulation, ACA, ICA, MCA, aneurysms of size, aneurysms of the neck, ruptured aneurysm, and SAH, were extracted from the manuscript text, patient demographic tables, and on-line tables and figures. STATA 15.1 was used to analyze the data. Odds ratio (OR; 95% Cl) was used to analyze the risk factors of no-reflow/slow-flow. Higgins index (I2) is used to evaluate heterogeneity. If the heterogeneity test is *P* ≥ 0.1 and I2 ≤ 50%, it indicates that there is homogeneity between studies, and the fixed effects model is used for combined analysis; if *P* < 0.1, I2 > 50%, it indicates that the study is heterogeneous, and we use sensitivity analysis to find the source of heterogeneity. If the heterogeneity is still large, we use the random-effects model or give up the combination of results and use descriptive analysis. A funnel plot was used to analyze publication bias.

## Results

### The Results of the Literature Search

**Figure 1:**
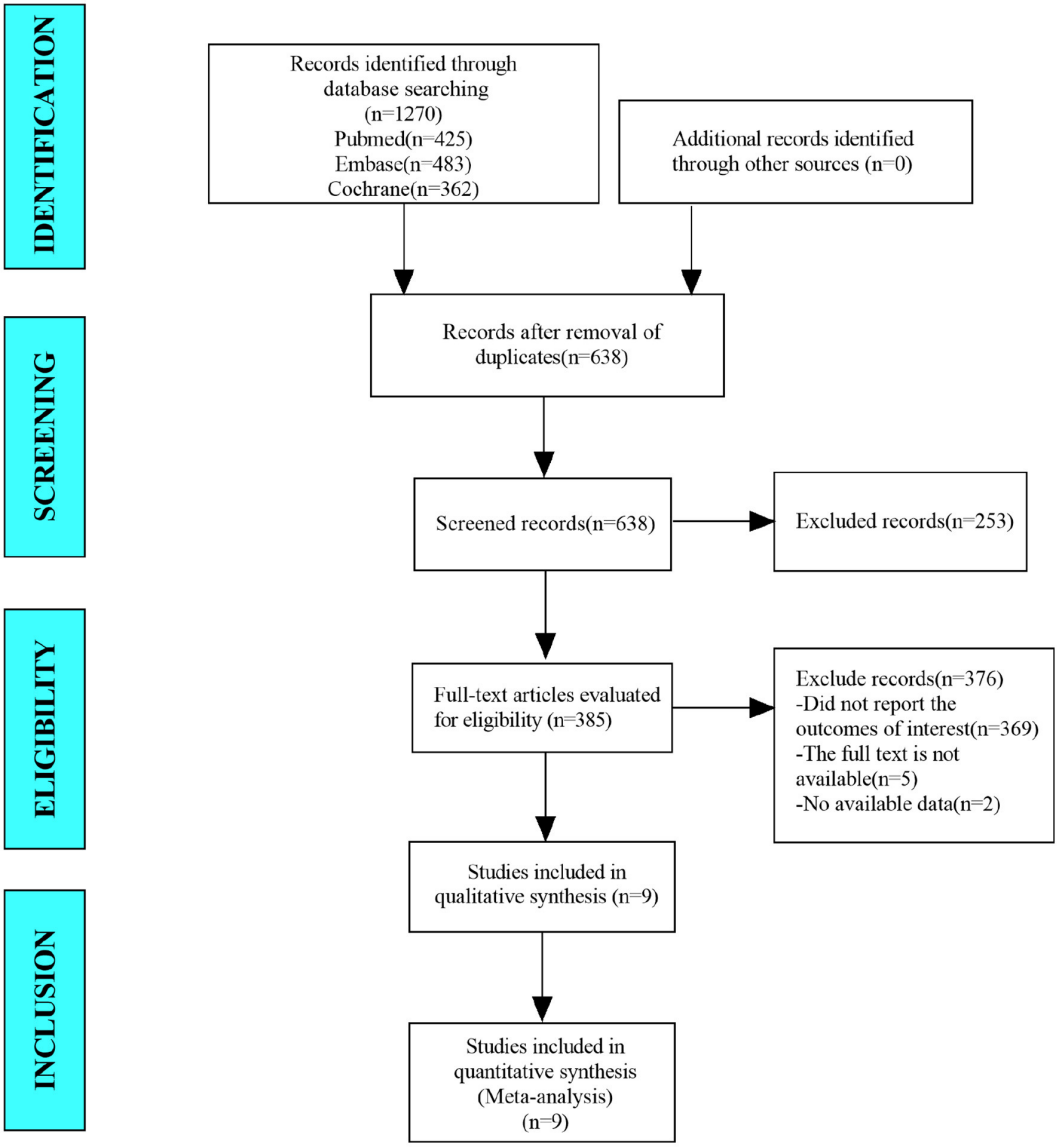
Flow diagram for the selection of studies.

In this study, a total of 1,270 studies were retrieved from the database. After eliminating duplicate studies, 638 were obtained. After browsing titles and abstracts, 385 studies were obtained. Finally, nine studies were meta-analyzed through full-text reading (Figure 1).

### Baseline Characteristics and Quality Assessment of the Included Studies

A total of nine retrospective studies were included in this meta-analysis. The sample size of patients with intracranial aneurysms ranged from 241 to 3,530, and a total of 9,532 patients were included in the present meta-analysis. Patients in four studies were from China, patients in two studies were from Korea, and the others were from Europe and America. The NOS score used for quality assessment is all above seven and meets the requirements. The baseline characteristics quality assessment of the included studies is shown in Table 1.

**Table 1:** Baseline characteristics and quality assessment of the included studies.

**References**	**Research type**	**Study area**	**Number of cases**	**Gender**	**Age**	**Follow-Up time**	**NOS score**
				**(Male/Female)**	**(Year)**	**(Month)**	
Tian et al. (12)	Retrospective	China	504	219/285	52.5 ± 10.7	13.6 ± 4.5	7
Choi et al. (7)	Retrospective	Korea	3,530	948/2,094	57.8 ± 10.9	21.4 ± 16.8	8
Futchko et al. (14)	Retrospective	USA	296	64/232	56.4	/	8
Mortimer (15)	Retrospective	UK	241	166/75	49.9 ± 12.5	31	7
Jeon (16)	Retrospective	Korea	870	275/595	57.9 ± 11.0	30.8 ± 8.3	7
Li (17)	Retrospective	China	1,335	467/868	54.0 ± 9.8	/	8
Zhang (18)	Retrospective	China	283	205/78	51.7 ± 8.9	/	7
Nishido (19)	Retrospective	France	1,815	986/829	50.5 ± 12.9	/	7
Huang (20)	Retrospective	China	658	243/415	/	/	8

### Results of Meta-Analysis

**Figure 2:**
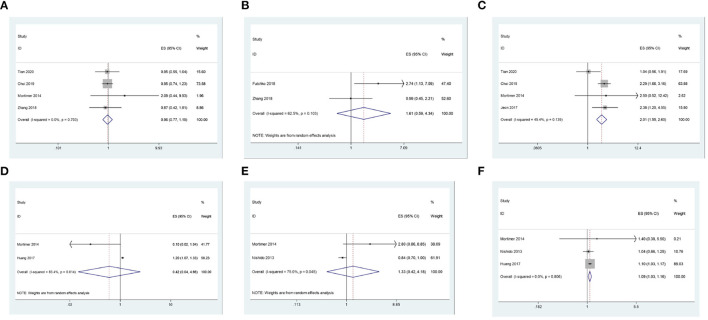
**(A)** The correlation between gender and recurrence after coil embolization for intracranial aneurysms. **(B)** The correlation between smoking and recurrence after coil embolization for intracranial aneurysms. **(C)** The correlation between posterior circulation and recurrence after coil embolization for intracranial aneurysms. **(D)** The correlation between ACA and recurrence after coil embolization for intracranial aneurysms. **(E)** The correlation between ICA and recurrence after coil embolization for intracranial aneurysms. **(F)** The correlation between MCA and recurrence after coil embolization for intracranial aneurysms.

We first explored the correlation between gender (female) and recurrence after coil embolization for intracranial aneurysms. There are 4 studies, including 4,558 patients, that reported the association between gender (female) and recurrence after coil embolization for intracranial aneurysms. Since there is no significant heterogeneity (I2 = 0.0%, *P* = 0.793 > 0.1), a meta-analysis was conducted through a fixed-effects model. The pooled results show that there is no significant association between gender (female) and recurrence after coil embolization for intracranial aneurysms (OR = 0.96, 95% CI: 0.77–1.19, *P* = 0.707 > 0.05; Figure 2A). We also pooled the results through a random-effects model (I2 = 62.5%, *P* = 0.103 > 0.1), thus we found that there is no significant association between smoking and recurrence after coil embolization for intracranial aneurysms (OR = 1.61, 95% CI: 0.59–4.34, *P* = 0.351 > 0.05; enrolling 579 patients; Figure 2B).

We continue to explore the correlation between the location of the aneurysm and the recurrence of intracranial aneurysms after coil embolization. There are four studies, including 5,145 patients, that reported the association between posterior circulation and recurrence after coil embolization for intracranial aneurysms. Since there is no significant heterogeneity (I2 = 45.4%, *P* = 0.139 > 0.1), a meta-analysis was conducted through a fixed-effects model. The pooled results indicate that intracranial aneurysms that occur in posterior circulation have a significantly higher risk of recurrence after coil embolization (OR = 2.01, 95% CI: 1.55–2.60, *P* = 0.000 < 0.05; Figure 2C). There are two studies, including 899 patients, that reported the association between ACA and its recurrence after coil embolization for intracranial aneurysms. Since there is significant heterogeneity (I2 = 83.4%, *P* = 0.014 < 0.1), a meta-analysis was conducted through a random-effects model. The polled results indicate that there is no significant association between ACA and recurrence after coil embolization for intracranial aneurysms (OR = 0.42, 95% CI: 44–4.66, *P* = 0.483.05; Figure 2D). Additionally, pooled results also show that there is no significant association between ICA and recurrence after coil embolization for intracranial aneurysms (OR = 1.33, 95% CI: 0.42–4.18, *P* = 0.627 > 0.05; enrolling 2,056 patients) from a random-effects model (I2 = 75.0%, *P* = 0.045 < 0.1; Figure 2E). However, pooled results show that intracranial aneurysms that occur in MCA have a significantly higher risk of recurrence after coil embolization (OR = 1.09, 95% CI: 1.03–1.16, *P* = 0.004 < 0.05; enrolling 2,714 patients) from a fixed-effects model (I2 = 0.0%, *P* = 0.806 > 0.1; Figure 2F).

**Figure 3:**
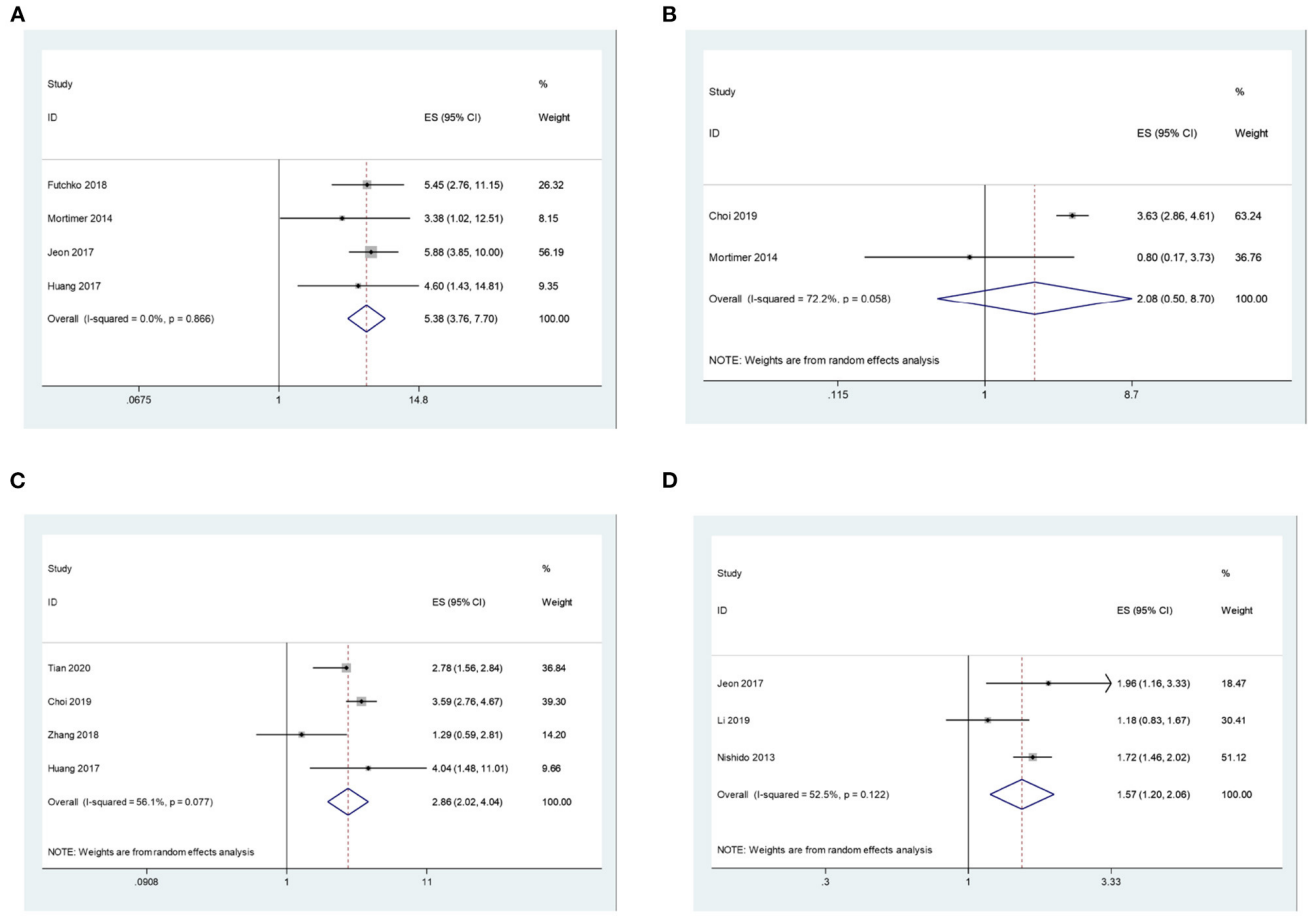
**(A)** The correlation between aneurysms of size > 7 mm and recurrence after coil embolization for intracranial aneurysms. **(B)** The correlation between aneurysms of neck > 4 mm and recurrence after coil embolization for intracranial aneurysms. **(C)** The correlation between ruptured aneurysm and recurrence after coil embolization for intracranial aneurysms. **(D)** The correlation between SAH and recurrence after coil embolization for intracranial aneurysms.

In addition, we explored the correlation between disease characteristics and recurrence after coil embolization. There are four studies, including 2,065 patients, that reported the association between aneurysms of size > 7 mm and their recurrence after coil embolization for intracranial aneurysms. Since there is no significant heterogeneity (I2 = 0.0%, *P* = 0.866 > 0.1), a meta-analysis was conducted through a fixed-effects model. The pooled results indicate that intracranial aneurysms of size > 7 mm have a significantly higher risk of recurrence after coil embolization. (OR = 5.38, 95% CI: 3.76–7.70, *P* = 0.000 < 0.05; Figure 3A). There are two studies, including 3,771 patients, that reported the association between aneurysms of neck > 4 mm and recurrence after coil embolization for intracranial aneurysms. Since there is significant heterogeneity (I2 = 72.2%, *P* = 0.058 < 0.1), a meta-analysis was conducted through a random-effects model. The pooled results indicate that there is no significant association between aneurysms of neck > 4 mm and recurrence after coil embolization for intracranial aneurysms (OR = 2.08, 95% CI: 0.50–8.70, *P* = 0.315 > 0.05; Figure 3B).

Lastly, pooled results show that ruptured aneurysm (OR = 2.86, 95% CI: 2.02–4.04, *P* = 0.000 < 0.05; enrolling 4,975 patients) and SAH (OR = 1.57, 95% CI: 1.20–2.06, *P* = 0.001 < 0.05; enrolling 4,020 patients) can all significantly increase the risk of recurrence after coil embolization with random effects models (I2 = 56.1%, *P* = 0.077 < 0.1; I2 = 52.5%, *P* = 0.122 > 0.1; Figures 3C,D).

### Publication Bias

The funnel plot of this study is shown in Supplementary Figure S1. The funnel plot is basically symmetrical, indicating that there is no obvious publication bias in this study.

### Sensitivity Analysis

Sensitivity analysis eliminates each included study one by one and performs a summary analysis on the remaining studies to assess whether a single included study has an excessive impact on the results of the entire meta-analysis. The results showed that none of the studies had an excessive impact on the results of the meta-analysis, indicating that the results of the remaining studies are stable and reliable.

## Discussion

Intracranial aneurysm is a prevalent cerebral disorder with severe injury and coil embolization has been identified as a prevalent therapeutic method in the intracranial aneurysm. In the present study, we identified the risk factors of recurrence of intracranial aneurysm after coil embolization using a meta-analysis based on nine studies.

In our meta-analysis, we found that there is no significant association between gender and smoking with recurrence after coil embolization for intracranial aneurysms. However, it has been reported that smoking is harmful to the clinical outcomes of intracranial aneurysm patients using coil embolization, and aneurysms are more prevalent in women than men (21). The influence of gender and smoking on the recurrence after coil embolization for intracranial aneurysms may be complicated and are affected by other factors. It has been identified that ACA, ICA, and MCA are close factors of intracranial aneurysms (22–27). Previous investigation has shown that posterior circulation is a risk factor for coil embolization of unruptured aneurysms (28). The behavior of posterior circulation has been proved to associate with PcoA aneurysms recanalization (29). Our analysis showed that MCA bifurcation and posterior circulation aneurysms presented a higher recurrence risk. It has been reported that endovascular treatment of wide-neck MCA and basilar apex aneurysms resulted in a core lab adjudicated Raymond Roy (14) occlusion rate of 30.6% and self-reported results at follow-up favor better angiographic outcomes (30). It suggests the necessity and significance of novel endovascular devices specifically designed to treat complex intracranial aneurysms.

Our data also revealed that ruptured aneurysm and SAH were positively correlated with the risk of recurrence after coil embolization. Recently, a study of the comparison between outcomes of endovascular and surgical treatments of ruptured anterior communicating artery aneurysms show that aneurysms with the first presentation of SAH secondary to a ruptured anterior communicating artery aneurysm treated by endovascular coiling have an increased risk of recurrence vs. those treated with clipping (31). These findings may provide some reference for treatment decisions of a multi-disciplinary team. It has been identified that the size of intracranial aneurysms significantly affects the selection and treatment effectiveness of intracranial aneurysms (32–35). Consistently, our analysis showed that intracranial aneurysms of size > 7 mm have a significantly higher risk of recurrence after coil embolization.

There are still some limitations in the current study. In this work, we provided a meta-analysis of the risk factors for recurrence of intracranial aneurysms after coil embolization. Despite the crucial risk of rebleeding or symptomatic recurrences needing retreatment in the model, we did not describe them in our analysis because there was no such detailed data in the literature. Meanwhile, the aspect of time and the aneurysms recurrence were not considered in the current study because there was no such detailed data in the literature, which is crucial for treatment decisions. In this study, we only included retrospective studies. Other types of studies should be considered in future investigations.

## Conclusions

This meta-analysis identified the characteristics of intracranial aneurysms with MCA, posterior circulation, size > 7 mm, ruptured aneurysm, and SAH as the risk factors of recurrence after coil embolization for intracranial aneurysms. Our finding enriches the understanding of the recurrence of intracranial aneurysms after coil embolization in patients, providing the theoretical reference for the clinical application of coil embolization for intracranial aneurysms. Meanwhile, it is crucial to design novel and specific endovascular devices for the treatment of these complex intracranial aneurysms and attenuate their recurrence.

## Data Availability Statement

The raw data supporting the conclusions of this article will be made available by the authors, without undue reservation.

## Author Contributions

GG, JJ, YR, and JZ designed the study and wrote the manuscript. GG, YR, BY, YWu, SW, YS, XW, and YWa performed the analysis. All authors contributed to the article and approved the submitted version.

## Funding

This study was supported by the Scientific Research Foundation of Shanxi Intelligence Institute of Big Data Technology and Innovation (SIBD-2020-YL0052).

## Conflict of Interest

The authors declare that the research was conducted in the absence of any commercial or financial relationships that could be construed as a potential conflict of interest.

## Publisher's Note

All claims expressed in this article are solely those of the authors and do not necessarily represent those of their affiliated organizations, or those of the publisher, the editors and the reviewers. Any product that may be evaluated in this article, or claim that may be made by its manufacturer, is not guaranteed or endorsed by the publisher.

## Supplementary Material

Supplementary Figure S1 Funnel plot for evaluating the publication bias of this meta-analysis.
