# Supplementary figures and images for: Risk Factors for Recurrence of Intracranial Aneurysm After Coil Embolization: A Meta-Analysis
Source: Front Neurol. 2022 Jul 22;13:869880. doi: 10.3389/fneur.2022.869880 (PMC9355382; doi:10.3389/fneur.2022.869880)

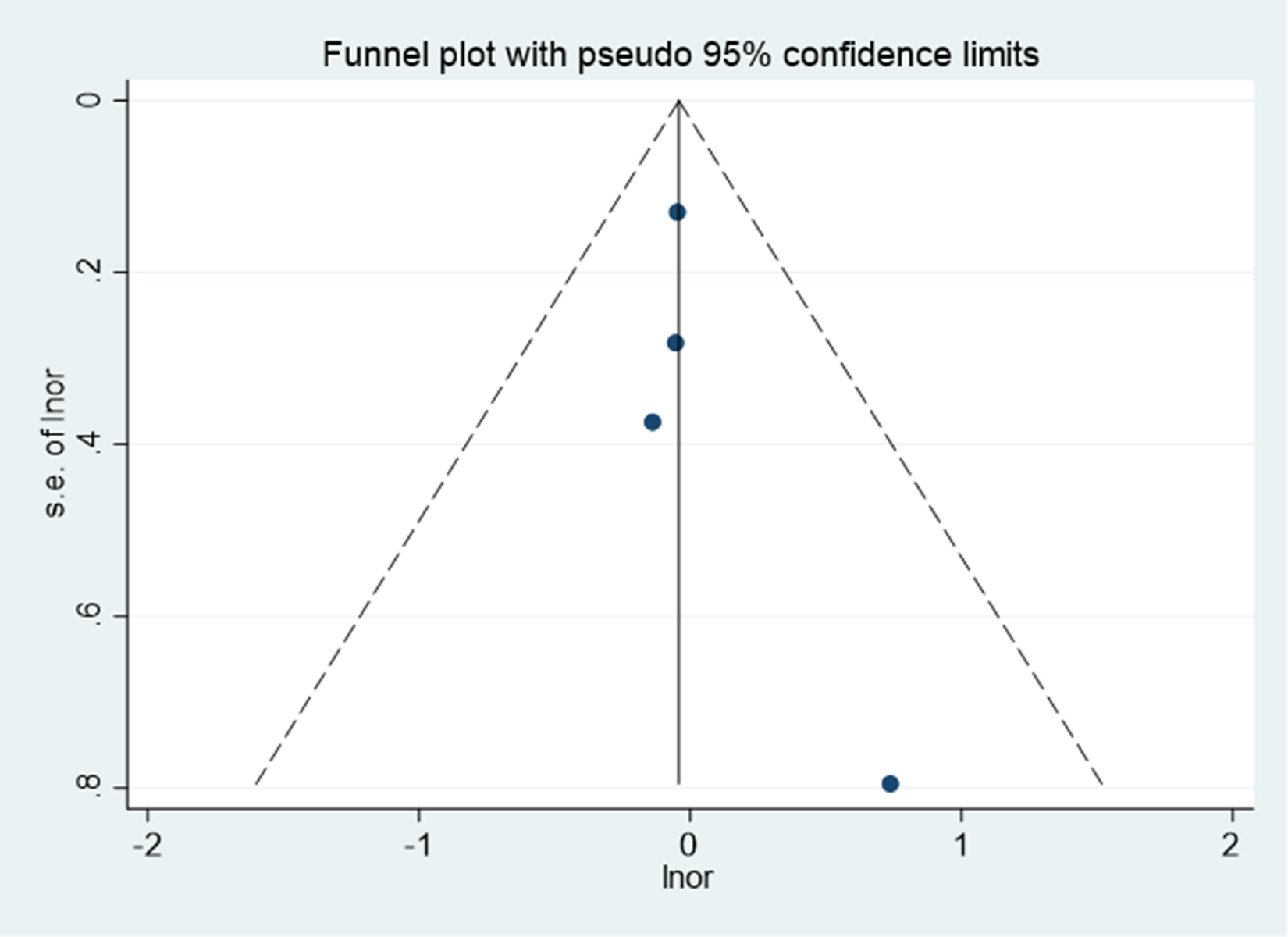

Supplement: Supplementary Figure S1 — Funnel plot for evaluating the publication bias of this meta-analysis. [file Image_1.tif]
